# Changes in the Subpopulations of Porcine Peripheral Blood Lymphocytes Induced by Exposure to Low Doses of Zearalenone (ZEN) and Deoxynivalenol (DON)

**DOI:** 10.3390/molecules21050557

**Published:** 2016-04-27

**Authors:** Michał Dąbrowski, Kazimierz Obremski, Magdalena Gajęcka, Maciej Tadeusz Gajęcki, Łukasz Zielonka

**Affiliations:** Department of Veterinary Prevention and Feed Hygiene, Faculty of Veterinary Medicine, University of Warmia and Mazury in Olsztyn, Oczapowskiego 13/29, 10-718 Olsztyn, Poland; michal.dabrowski@uwm.edu.pl (M.D.); obremski@uwm.edu.pl (K.O.); mgaja@uwm.edu.pl (M.G.); gajecki@uwm.edu.pl (M.T.G.)

**Keywords:** deoxynivalenol, zearalenone, pig, flow cytometry, lymphocytes CD4^+^8^−^, CD4^−^8^+^ and CD4^+^8^+^

## Abstract

Zearalenone and deoxynivalenol are secondary metabolites of fungi of the genus *Fusarium*. The presence of mycotoxins in cereals and the resulting contamination of feeds and foods pose health risks for animals and humans. The dangers associated with high doses of mycotoxins have been extensively researched but very little is known about NOAEL (No Observed Adverse Effect Level) doses or exposure to a combination of mycotoxins (mixed mycotoxicoses). The aim of this study was to determine the effects of six-week exposure to NOAEL doses of individual and combined mycotoxins on the subpopulations of CD4^+^8^−^, CD4^−^8^+^ and CD4^+^8^+^ lymphocytes in the peripheral blood of pigs. The experiment was performed on 72 gilts with average body weight of 25 kg, divided into three experimental groups (E1, E2 and E3, administered zearalenone (ZEN), deoxynivalenol (DON) and ZEN + DON, respectively, on a daily basis) and a control group (C) receiving placebo. Changes in lymphocyte subpopulations were evaluated by flow cytometry at weekly intervals (experimental days 7, 14, 21, 28, 35 and 42). A linear increase in the percentage of CD4^+^8^+^ lymphocytes was highly correlated with time (*r* = 0.682) in group C. The correlations and linear increase in the above subpopulation were disrupted in the remaining groups. In group E3, a statistically significant (*p* < 0.05) decrease in CD4^+^8^+^ counts was observed in week 5, which could point to a transient depletion of regulatory mechanisms of immune responses. The noted results also suggest that in mixed mycotoxicosis, ZEN and DON exerted stronger immunomodulatory effects.

## 1. Introduction

Mycotoxins are secondary metabolites of fungi which can cause infections known as mycotoxicoses in humans and animals. According to the Food and Agriculture Organization of the United Nations (FAO), more than 25% of cereals produced globally are contaminated with mycotoxins [1]. One of the leading contributors to the spread of fungal infections in crops is no-till farming [2] which significantly reduces cultivation costs in large-area farms. Mycotoxins are produced mainly during the growing season, but if harvested crops are stored under inadequate conditions, including in environments with high moisture levels, mycotoxins can also be produced outside the growing season [3]. Fungi of the genus *Fusarium* are prevalent crop pathogens in temperate climates of Northern America, Europe and Asia [4] Fusarium mycotoxins are low-molecular-weight compounds, which are produced in response to environmental stressors and are toxic for humans and animals [5]. Zearalenone, fumonisin B_1_ (FB_1_) and trichothecenes, in particular deoxynivalenol (DON) and nivalenol (NIV), are the most prevalent and toxic compounds for animals [6]. *Fusarium* mycotoxins are characterized by a broad spectrum of toxic activity, and they act as immunomodulators in humans and animals [7].

Deoxynivalenol is a mycotoxin commonly identified in wheat, maize and barley [8]. The toxic effect of DON on animals and humans has been recently reviewed [9,10,11,12]. It is characterized by high tropism for intestinal epithelial cells and immune system cells with a high division potential [13]. Exposure to high doses of DON can lead to diarrhea, vomiting, leukocytosis, gastrointestinal bleeding and, ultimately, death. Low doses contribute to anorexia, lower weight gains and lower feed efficiency [14]. Immune system cells targeted by DON include monocytes, macrophages and lymphocytes [15]. Subject to dose, this mycotoxin can have immunosuppressive or immunostimulatory effects. In mice, high doses of DON led to rapid leukocyte apoptosis [16], whereas low doses increased cytokine levels and serum IgA concentrations [17]. At the cellular level, DON binds with ribosomal peptidyl-transferase and inhibits protein synthesis. The mycotoxin also binds with eukaryotic ribosomes to provoke a ribotoxic stress response, which is responsible for the phosphorylation of mitogen-activated protein (MAP) kinases: ERK1/2, JNK and p38 [18]. Active MAP kinases activate transcription factors which regulate cell proliferation, differentiation, apoptosis and inflammatory processes. Immunosuppression induced by DON decreases the activity of T and B cells and lowers the production of antibodies [19].

Zearalenone is an estrogen-like nonsteroidal mycotoxin with a macrocyclic lactone ring fused to resorcylic acid [20]. In a study by Placinat *et al.* [21], wheat, barley, oats, rye and feeds produced in Europe contained up to 8 mg/kg of zearalenone (ZEN). Exposure to ZEN lowers fertility and litter size, leads to changes in the size of adrenal glands, thyroid gland, pituitary gland, as well as changes in serum progesterone and estrogen levels [22]. Orally administered ZEN is metabolized to alpha- and beta-zearalenol (α-ZEL, β-ZEL) mainly in the liver [23]. Alpha-ZEL has stronger affinity for the estrogen receptor than ZEN and β-ZEL [24]. Salem [25] demonstrated that estrogen receptors are present on different immunocompetent cells and that the immune system is sensitive to the presence of estrogen-like compounds. The existing research focuses on ZEN’s effect on the reproductive system, and very few studies are dedicated to its influence on the immune system. Marin *et al.* [26,27] demonstrated that ZEN and its derivatives have a varied effect on innate immunity parameters by modulating the levels of proinflammatory cytokines in peripheral blood cells. In a study by Obremski [28], ZEN doses of up to 8 µg/kg body weight (BW) induced changes in cytokine secretion by Peyer’s patch lymphocytes stimulated with lipopolysaccharides (LPS).

Recent studies revealed that small amounts of mycotoxins are ubiquitous in cereals [29]. The health implications of exposure to several mycotoxins are difficult to define based on the toxic properties of individual mycotoxins. Various types of interactions take place between mycotoxins, including synergistic, additive, subadditive and antagonistic [30]. There is a growing body of research into combined exposure to several mycotoxins, with special focus on the effects of multi-contamination on immunological, morphological and reproductive parameters in humans and animals. Girish *et al.* [31] demonstrated that feeds naturally contaminated with DON, ZEN, 15-acetyldeoxynivalenol (15-Ac-DON) and HT-2 provoked changes in T and B cell populations in the small intestine of turkeys and disrupted enterocyte proliferation in ileal crypts. In an *in vivo* experiment conducted on 24-week-old pigs, combined administration of FB_1_ and DON induced more pronounced histopathological changes in the liver, lungs and kidneys than when those mycotoxins were administered individually. The same study also revealed that co-contamination with FB_1_ and DON led to a more significant decrease in splenic *m*RNA levels of IL-8, IL-1beta and IL-6 in experimental animals [32]. In an *in vivo* study of mice, Ren *et al.* [33] demonstrated that ZEN and DON synergistically deregulated the serum levels of IL-1, IL-4 and C3 in experimental animals. 

The objective of this study was to evaluate the influence of low doses of two co-occurring mycotoxins, ZEN and DON [34], on CD4^+^8^−^; CD4^+^8^+^ and CD4^−^8^+^ counts in pigs during six-week exposure to the analyzed mycotoxins administered *per os* individually and in combination.

## 2. Results

Changes in the percentages of CD4^+^8^−^, CD4^+^8^+^ and CD4^−^8^+^ lymphocytes in every week of the experiment, expressed as arithmetic means and standard deviation, are presented in Table 1.

**Figure 1:**
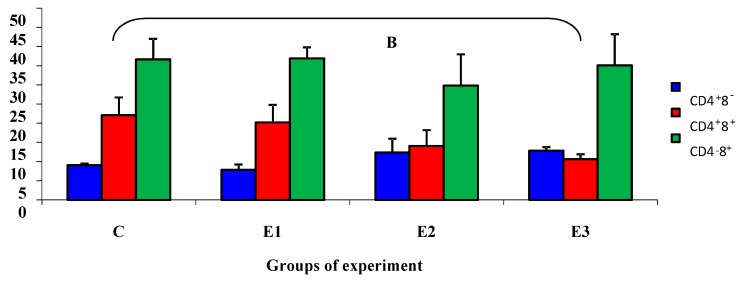
The percentage of individual subpopulation of lymphocytes in the fifth week of the experiment.

Statistically significant differences (*p* ≤ 0.05) were noted in the subpopulation of CD4^+^8^+^ cells. Differences were observed between group C and group E3 in week 5 (Figure 1).

**Figure 2:**
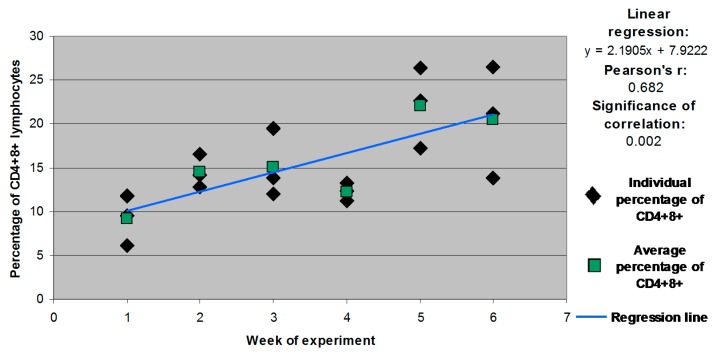
Individual and mean percentages of CD4^+^8^+^ lymphocyte subpopulations in group C.

Statistically significant differences (*p* ≤ 0.05) in CD4^+^8^+^ counts were also found in group C between week 1 and weeks 5 and 6. A significant correlation was observed between the size of the CD4^+^8^+^ subpopulation and the animals’ age in group C. The correlation coefficient was determined at *r* = 0.682 at a significance level of *p* = 0.002 (Figure 2).

**Figure 3:**
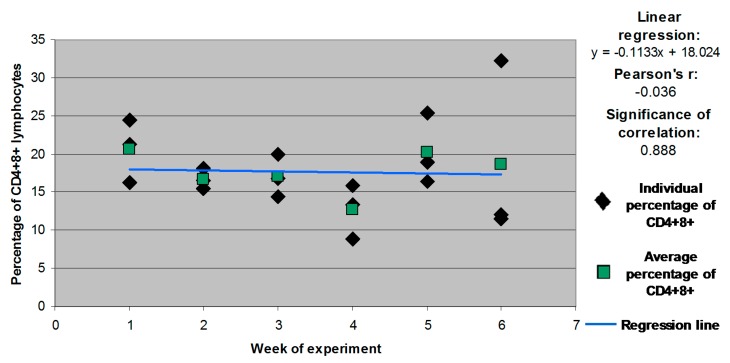
Individual and mean percentages of CD4^+^8^+^ lymphocyte subpopulations in group E1.

**Figure 4:**
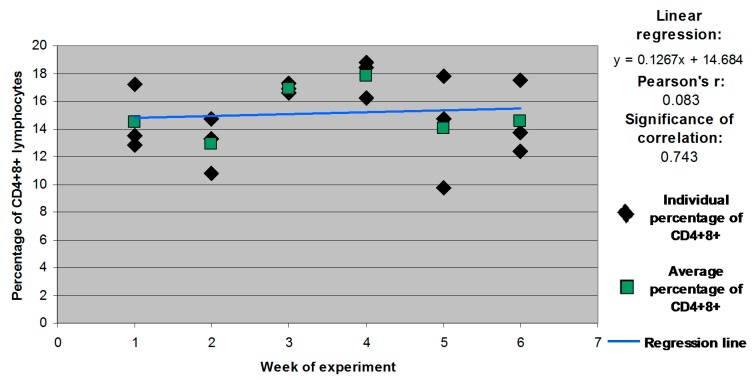
Individual and mean percentages of CD4^+^8^+^ lymphocyte subpopulations in group E2.

**Figure 5:**
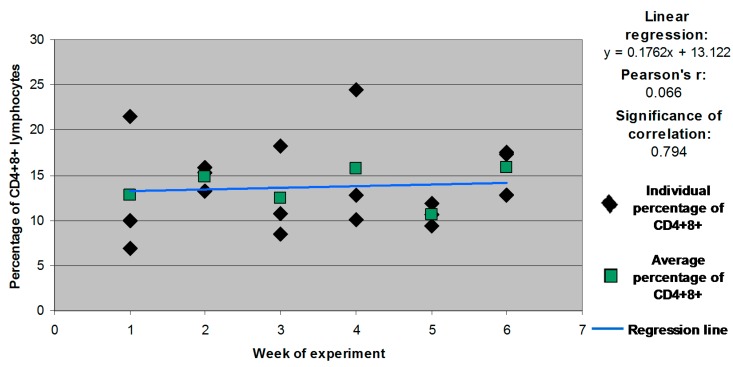
Individual and mean percentages of CD4^+^8^+^ lymphocyte subpopulations in group E3.

Statistically significant differences and correlations were not noted in the remaining weeks or groups (Figure 3, Figure 4 and Figure 5 for groups E1, E2 and E3, respectively).

## 3. Discussion

The aim of this experiment was to evaluate the *in vivo* effect of low doses of ZEN and DON, administered individually or in combination, on the function of the immune system based on the subpopulations of CD4^+^8^−^, CD4^+^8^+^ and CD4^−^8^+^ lymphocytes in the peripheral blood of pigs.

The results revealed that long-term exposure to low doses of ZEN, DON and ZEN + DON disrupted linear proliferation of CD4^+^8^+^ cells. Co-contamination of feed with both mycotoxins had a stronger effect on the immune system and led to a transient decrease in the percentage of CD4^+^8^+^ lymphocytes in week 5 of exposure.

The *in vivo* effects of various mycotoxins, in particular ZEN and DON, on the size of different lymphocyte subpopulations have been insufficiently documented in the literature. In a study by Swamy *et al.* [35], a cytometric analysis performed after 22 days of continuous administration of feed naturally contaminated with DON (5.8 mg/kg), fusaric acid (49.3 mg/kg), ZEN (0.5 mg/kg) and 15-Ac-DON (0.3 mg/kg) revealed that the analyzed mycotoxins did not influence CD4^+^8^−^, CD4^+^8^+^ and CD4^−^8^+^ counts. Ferrari *et al.* [36] did not report statistically significant differences in the size of CD3^−^8^+^, CD4^+^8^−^, CD4^−^8^+^, CD4^+^8^+^ and TCR γδ^+^ subpopulations between the control group and the experimental group administered DON at 1 mg/kg of feed. Goyarts *et al.* [37] administered feed contaminated with DON (5.7 mg/kg of feed) to experimental animals and did not observe changes in the percentages of lymphocytes and granulocytes or hematocrit levels. Based on their results, they concluded that hematological parameters are poor indicators of immune disorders caused by DON. Different results were reported by Levkut *et al.* [38] who evaluated the effect of feed co-contamination with DON (3.4 and 8.2 mg/kg of feed) and ZEN (3.4 and 8.3 mg/kg of feed) on lymphocyte counts in peripheral blood and duodenal epithelium of broiler chickens. They demonstrated that exposure to mycotoxins lowered the size of CD3^+^ subpopulations and inhibited the phagocytic activity of white blood cells in peripheral blood. Rotter *et al.* [39] also demonstrated that exposure to DON (4 mg/kg of feed) influenced blood morphology and biochemistry. Their experimental design was similar to that presented in our study, and they evaluated blood parameters at weekly intervals over a period of six weeks. The cited authors observed a transient decrease in total protein and beta-globulin levels in the blood serum. The results noted in this study indicate that frequent tests are much more useful in analyzing the risk of exposure to DON because the body adapts to the mycotoxin, and the analyzed parameters return to control group levels over time. 

In this study, changes were noted in the subpopulation of lymphocytes with the CD4^+^8^+^ phenotype. Those cells are referred to as double-positive (DP) lymphocytes, and their percentage in porcine peripheral blood increases with age, from 8% to 64% of the total T cell count [40]. The present experiment demonstrated that in animals not exposed to mycotoxins (group C), the increase in the percentage of DP lymphocytes was strongly linearly correlated with time. In the remaining groups, significant variations were noted in the size of the analyzed subpopulation which was not correlated with the animals’ age. Double-positive lymphocytes are effector memory cells. Zuckermann and Husmann [41] isolated DP lymphocytes from pigs vaccinated against Aujeszky’s disease and subjected them to secondary stimulation with the vaccine antigen, which resulted in strong proliferation and secretion of IFNγ. Kaser *et al.* [42] demonstrated that most lymphocytes with the Foxp3 marker of T regulatory cells (Forkhead-box p3) belong to the population of DP lymphocytes. The results of our experiment suggest that even low mycotoxin doses can lead to immune disorders, thus affecting the immune status of a herd. 

In week 5, the subpopulation of DP lymphocytes decreased in group E3 relative to group C. This observation indicates that feed co-contaminated with DON + ZEN exerts a stronger immunosuppressive effect than feed containing only one mycotoxin. The interactions between ZEN and DON have been documented by relatively few studies. In an *in vitro* study analyzing the combined contribution of *Fusarium* mycotoxins to myelotoxicity, Ficheux *et al.* [43] observed the presence of additive interactions between ZEN and DON. Chen *et al.* [44] found that co-contamination with ZEN + DON lowered serum total protein, albumin and globulin in an *in vivo* study of pigs. The analyzed mycotoxins enhanced the activity of gamma-glutamyltransferase, aspartate aminotransferase and alanine aminotransferase. In our experiment, the decrease in the subpopulation of DP lymphocytes was transient, and CD4^+^8^+^ percentages returned to group C levels in week 6. A similar two-phase effect was reported by Pinton *et al.* [45] who investigated the influence of DON on lymphocyte proliferation in animals immunized with ovalbumin. The proliferative activity of lymphocytes obtained from animals exposed to the mycotoxin was higher on day 21, but lower between days 35 and 49 of the experiment, relative to the control group. The neutral red assay performed on IPEC-1 and IPEC-J2 cells also demonstrated similar results. Cell viability was lowered after 24 h of incubation with 200 ng/mL of DON, but it was restored to control group levels after 48 and 72 h of incubation [46].

This study was performed on pigs, an animal species most susceptible to ZEN and DON [22,47]. The structure and functions of gastrointestinal and immune systems are highly similar in pigs and humans [48,49]; therefore, the results of pig studies are often used in analyses of human diseases. The applied doses of ZEN and DON are similar to mycotoxin concentrations found in food products. In a study by Shahzad *et al.* [50], ZEN levels in wheat-based products, mainly pasta, were determined at 69.8 µg/kg. In a study of bread contamination with trichothecenes, DON levels were estimated at 146.6 µg/kg [51].

Mycotoxins are frequently identified in popular food products, and prolonged exposure to toxins can increase the risk of health problems. The results of studies confirming the presence of low doses of ZEN and DON in foods and feeds should be used to verify the maximum permissible levels of mycotoxins to guarantee the biological safety of the food chain. Further research into the effects of low dose contamination with ZEN and DON on the immune system should focus on the function of DP lymphocytes. 

## 4. Materials and Methods

All experimental procedures involving animals were carried out in compliance with Polish regulations setting forth the terms and conditions of animal experimentation (decision No. 88/N of 16 December 2009 of the Local Ethics Committee for Animal Experimentation).

### 4.1. Animals

The experiment was performed in the Department of Veterinary Prevention and Feed Hygiene at the Faculty of Veterinary Medicine of the University of Warmia and Mazury in Olsztyn on 72 clinically healthy gilts purchased from a pig breeding farm in Dobrzejewice. The animals were Polish Landrace x Polish Large White crossbreeds with average initial body weight of 25 kg. Pigs were kept in group pens with free access to water, and were fed in the morning and evening under a restrictive feeding system. Feed was analyzed for the presence of ZEN, DON and ochratoxin (OTA) with the use of high-performance liquid chromatography (HPLC) Agilent, type 1100, coupled with a diode array detector (DAD) and a fluorescence detector (FLD). The obtained values did not exceed the limits of quantitation (LoQ) of 2 ng/g for ZEN, 5 ng/g for DON and 2 ng/g for OTA.

### 4.2. Experimental Design

The animals were randomly divided into three experimental groups (E1, E2 and E3) and one control group (C) of 18 pigs each. Group E1 pigs were orally administered ZEN at 40 µg/kg BW, group E2 animals were orally administered DON at 12 µg/kg BW, and group E3 pigs were orally administered ZEN + DON at 40 and 12 µg/kg BW, respectively. Control group pigs were orally administered placebo. The analyzed mycotoxins and placebo were administered daily in gel capsules immediately before the morning feeding. The placebo capsule was filled with a control medium, and the experimental capsule contained mycotoxins. The experiment lasted six weeks (42 days). Animals from each group were weighed at weekly intervals. The average weight was used to determine mycotoxin doses which were combined with feed in the following week. The experimental procedure involving animals was identical to that applied by Zielonka *et al.* [52]

### 4.3. Reagents

Zearalenone and DON were synthesized and standardized in the Department of Chemistry at the Poznań University of Life Sciences. Zearalenone was biosynthesized on a rice grains medium with the use of *Fusarium graminearum* and *Fusarium culmorum*. Dried and ground rice grains were degreased with hexane. Mycotoxins were extracted with ethanol to prevent small amounts of the analyzed compounds from reaching the organic layer. Degreased material was acidified to pH 3, and ZEN was extracted several times with chloroform. Each time, the chloroform fraction was evaporated to near dryness. Condensed chloroform layers were combined with ethanol, evaporated to dryness and dissolved in 300 mL of water. Zearalenone was extracted from the aqueous layer with benzene and evaporated to dryness. It was purified in a chromatographic column filled with Kieselgel. Metabolites were eluted from the column with a 9:1 mixture of benzene and acetone, evaporated to dryness and dissolved in ethanol. The ethanol fraction was purified with heptane several times. Zearalenone was extracted from the condensed fraction with alcohol. The purity of the extracted mycotoxin was checked spectroscopically, and it was comparable to that of the Sigma-Aldrich (Saint Louis, MO, USA) ZEN standard.

Deoxynivalenol was biosynthesized with the use of *Fusarium graminearum* and *Fusarium culmorum* on rice grains. After four weeks of incubation, dried and ground rice grains were subjected to extraction with a 3:1 mixture of methanol and water. Deoxynivalenol was extracted by liquid-liquid partitioning with ethyl acetate, it was evaporated to dryness and dissolved in chloroform. The sample was dried, suspended in ethyl acetate and transferred to a column filled with a mixture of activated charcoal and Kieselgel. Deoxynivalenol was eluted from the column with ethyl acetate, and its content in each fraction was determined by HPLC.

The extracted mycotoxins were dissolved in 500 ml of ethanol (96% ethanol, SWW2442-90, Polskie Odczynniki Chemiczne SA, Gliwice, Poland). The obtained solutions were transferred to gelatin capsules to produce ZEN doses of 40 µg/kg BW and DON doses of 12 µg/kg BW. Capsules were incubated for 12 h at room temperature, in darkness, to evaporate the solvent. 

### 4.4. Blood Samples

Blood was sampled on experimental days 7, 14, 21, 28, 35 and 42 from three randomly selected pigs from each group. The animals were anesthetized with sodium thiopental during blood sampling. Peripheral blood was sampled from surgically exposed external jugular vein (*vena jugularis externa*). Blood was collected into polypropylene tubes with EDTA:K_3_ anticoagulant (Sarstedt AG & Co, Nümbrecht, Germany) sprayed on the inner surface.

### 4.5. Determination of the Size of T Cell Subpopulations

Blood samples of 50 µL were transferred to cytometry tubes and combined with monoclonal antibodies in the amount recommended by the manufacturer. Mouse Anti Pig (AbD Serotec, Kidlington, UK) antibodies specific for surface receptors CD4 (CD4a:FITC, MIL17 clone), CD8 (CD8a:PE, MIL-12 clone) and T cells were used. Blood samples were incubated on ice for 30 min. After incubation, red blood cells were lysed with the FACS^TM^ lysing solution (BD, San Jose, CA, USA) for 12 min at room temperature, in darkness. The remaining cells were rinsed with PBS and centrifuged at 300× *g* for 5 min at room temperature. Data were acquired with the use of the FACSCanto II flow cytometer (BD, San Jose, CA, USA) by recording 30,000 events for each sample. Lymphocytes were immunophenotyped in FACSDiva Software 6.1.3 (BD, San Jose, CA, USA).

### 4.6. Statistical Analysis

Changes in the percentage of CD4^+^8^−^, CD4^−^8^+^ and CD4^+^8^+^ subpopulations in the peripheral blood of pigs were expressed as mean values (±) and standard deviation (SD) for each group in every week of the experiment. Data were analyzed in the Statistica application (StatSoft Inc., St. Tulsa, OK, USA). In view of the administered doses of ZEN and DON and the period of administration, mean values were compared by one-way ANOVA with non-repeated measures. The equality of variances between groups, a mandatory procedure in ANOVA, was checked by the Brown–Forsythe test. Differences between groups were determined by Tukey’s honest significant difference (HSD) test at *p* ≤ 0.05 and *p* ≤ 0.01.

The presence of linear relationships between the animals’ age and the size of lymphocyte subpopulations was determined by calculating Pearson’s correlation coefficient. Data were analyzed in the Statistica application (StatSoft Inc.). The results were regarded as statistically significant at *p* ≤ 0.05.

## 5. Conclusions

The results of this study give rise to the following conclusions:

- Double-positive lymphocytes (CD4^+^8^+^) are most susceptible to ZEN and DON contamination;
- Low doses of ZEN and DON can disrupt the proliferation of double-positive lymphocytes (CD4^+^8^+^) and negatively affect the immune status of a herd;
- Co-contamination with ZEN+DON has a more toxic effect than exposure to individual mycotoxins, and it leads to a transient decrease in DP lymphocyte counts;
- Further research into the effects of low-dose contamination with ZEN and DON should focus on the function of DP lymphocytes and the consequences of a decrease in their subpopulations.

## Figures and Tables

**Table 1:** Average percentages of lymphocyte subpopulations + SD.

Group	Week of the Experiment	CD4^+^8^−^	SD	CD4^+^8^+^	SD	CD4^−^8^+^	SD
C	1	10.233	6.785	9.133 ^a^	2.867	32.866	20.947
	2	17	5.185	14.5	1.868	32.833	2.836
	3	11.1	1.969	15.1	3.915	32.4	6.158
	4	13.9	5.828	12.233	1.001	33	9.18
	5	9.066	0.404	22.066 ^a,b^	4.623	36.666	5.342
	6	13.566	2.409	20.5 ^a^	6.378	29.6	1.967
E1	1	9.966	7.755	20.6	4.132	38.966	11.403
	2	11.133	5.229	16.666	1.357	30.166	2.983
	3	14.366	2.0550	17.066	2.809	35.733	2.358
	4	10.833	0.896	12.666	3.557	36	4.853
	5	7.833	1.365	20.2	4.590	36.9	2.882
	6	10.533	1.921	18.566	11.809	29.833	5.75
E2	1	8.466	3.695	14.5	2.364	31.433	3.234
	2	12.266	1.6258	12.933	1.975	34.266	5.783
	3	14.033	2.891	16.933	0.351	32.5	6.755
	4	8.066	3.789	17.8	1.4	47.2	16.629
	5	12.333	3.636	14.066	4.086	29.8	8.150
	6	13.733	2.837	14.533	2.65	31.966	6.469
E3	1	9.766	0.7571	12.8	7.692	31.233	12.182
	2	15.133	1.778	14.833	1.361	34.766	5.832
	3	10.066	1.847	12.5	5.068	32.833	11.657
	4	11.766	3.181	15.766	7.5975	28.833	12.828
	5	12.8	0.9539	10.633 ^b^	1.2503	35.066	8.146
	6	13.8	4.330	15.9	2.688	26.5	6.908

^a^ Statistically significant differences within the group (*p* ≤ 0.05). ^b^ Statistically significant differences between the groups (*p* ≤ 0.05).
